# Supplementary material for: Mechanism of replication origin melting nucleated by CMG helicase assembly
Source: Nature. 2022 Jun 15;606(7916):1007–14. doi: 10.1038/s41586-022-04829-4 (PMC9242855; doi:10.1038/s41586-022-04829-4)
Supplement: Supplementary file 1 — This file contains Supplementary Figure 1; Supplementary Tables 1–3; legends for Supplementary Videos 1–5 and Supplementary References. [file 41586_2022_4829_MOESM1_ESM.pdf]

nature portfolio

https://doi.org/10.1038/s41586-022-04829-4

Supplementary information

Mechanism of replication origin melting nucleated by CMG helicase assembly

In the format provided by the authors and unedited

SI Guide

Mechanism of replication origin melting nucleated by CMG helicase assembly

Jacob S. Lewis[1], Marta H. Gross[2,5], Joana Sousa[1,4,5], Sarah S. Henrikus[1], Julia F. Greiwe[1], Andrea Nans[4], John F. X. Diffley[2] and Alessandro Costa[1,5]

[1] Macromolecular Machines Laboratory, The Francis Crick Institute, London, U.K.

[2] Chromosome Replication Laboratory, The Francis Crick Institute, London, U.K.

[3] Structural Biology Science Technology Platform, The Francis Crick Institute, London, U.K.

[4] Current affiliation: UCB Pharma, Slough, U.K.

[5] These authors contributed equally.

Table of contents

Supplementary Information
Supplementary References
Supplementary Figure 1
Supplementary Table 1
Supplementary Table 2
Supplementary Table 3
Supplementary Video 1
Supplementary Video 2
Supplementary Video 3
Supplementary Video 4
Supplementary Video 5

Supplementary Discussion

From the end of mitosis and through G1 phase, catalytically inactive MCM double hexamers (DHs) are loaded onto duplex DNA[7,8]. Upon entry into S phase, the pre-initiation complex is formed, when a set of firing factors are recruited to promote the binding of GINS and Cdc45 to MCM[9–11], in a process that requires Pol epsilon[33]. Pre-initiation complex formation depends on phosphorylation by the DDK[11,13–15] and CDK kinases[11,12,16]. Upon ADP release and ATP binding by MCM[1], we find in this study, a double CMG Pol epsilon (dCMGE) complex is formed. This leads to the reconfiguration of the DH interface, with the formation of a splayed dimer that pivots around the Mcm6 N-terminal domain. In this configuration, 1.5 turns of DNA double helix are exposed between the two rings, while nucleation of DNA melting is triggered symmetrically inside each MCM central channel. Within the helicase ring, DNA melting is promoted upon ATP-triggered untwisting of 0.7 turns of the double helix[1], in particular through the action of Mcm2 that pushes on the lagging strand while also pulling on the leading strand. As the minor grove is widened and DNA untwisted, Watson/Crick base pairs are broken, and three orphan bases are stabilised by an Mcm6-specific insertion of the N-terminal pore loop (Mcm6 wedge). At the same time, the DNA entrapped within the two helicase complexes remains in its original twisted configuration. Thus, the ATP-triggered structural change within the MCM motor does not promote the accumulation of torsional strain focused on the DNA found between the two helicases. This is possible because, unlike within the ATPase tier, no protein-DNA contact is maintained within the N-terminal tier upon DH-to-dCMGE conversion. As a result, the dimerization interface formed by the two N-terminal tiers within the DH changes register upon DH-to-dCMGE transition, through a 1-subunit rotation. It is this register shift in MCM that prevents any topological strain to accumulate within the

two MCM hexamers, while likely diffusing outside of the dCMGE complex. Our observation predicts that duplex DNA untwisting inside the MCM channel could occur in the context of one single CMGE complex. Biochemical work from the Bell and Gelles laboratories supports this notion[62]. In fact, an MCM variant that can be loaded onto DNA but not form a DH can support CMG formation and duplex DNA untwisting to wild type levels. The same variant however fails to support replication fork establishment, indicating that two symmetry related MCM hexamers are required to promote origin firing[62,63]. During this process, one strand is ejected from each CMG and becomes the translocation strand of the opposed CMG, in a process that causes the two helicases that cross paths[1]. Although this event is not understood at the molecular level, our discovery of the dCMGE intermediate that nucleates origin DNA melting invites a model for origin firing. In downstream events towards replication fork establishment, we speculate, unwinding of the intervening duplex DNA might require N-terminal interactions with duplex DNA to be maintained after dCMGE formation. Through the establishment of such N-terminal topological link, any new relative rotation of two separated dCMGE complexes would result in unwinding of the intervening duplex DNA. The lagging-strand engaging N-terminal Mcm6 wedge insertion, established upon dCMGE formation, could form this topological link. In a later step, replication forks are established upon dCMGE engagement by Mcm10, which triggers ATP hydrolysis by the CMG and lagging strand ejection, allowing the bypass of the two activated replicative helicases[1]. How Mcm10 functions during origin firing is not understood at the molecular level. Nucleoprotein transitions leading to origin activation are depicted in **Extended Data Fig. 8**.

Supplementary References

62. Champasa, K., Blank, C., Friedman, L. J., Gelles, J. & Bell, S. P. A conserved Mcm4 motif is required for Mcm2-7 double-hexamer formation and origin DNA unwinding. *Elife* 8, e45538 (2019).

63. Langston, L. D. & O'Donnell, M. E. An explanation for origin unwinding in eukaryotes. *Elife* 8, e46515 (2019).

Source Data

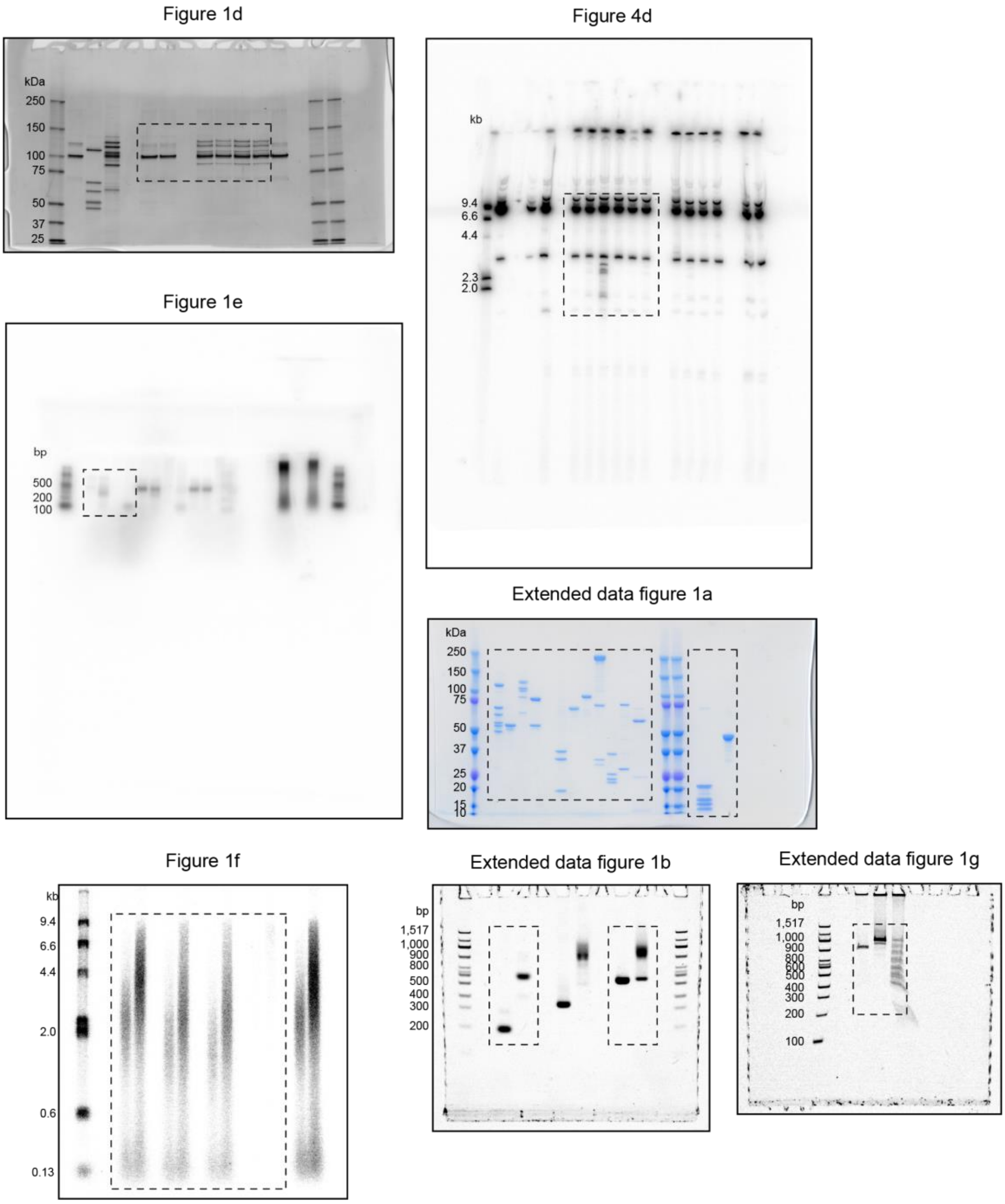

Supplementary Figure 1: Source images for all data obtained by electrophoretic separation in the Figures indicated.

Supplementary Table 1: Oligonucleotides and gene blocks used in this study.

Name	Usage	Sequence
gbJL004	Template for RFC for construction of pJL003	ATGGGCAGCAGCTGGTCTCACCCCCAATTTGAGAAAGGCGGTGGCAGCGGCGGCGGTAGCGGAGGTGGCAGCTGGTCACACCCACAATTCGAGAAAGAAAACCTGTACTTCCAATCCATGGGTTAC
oJL009	Forward primer for generation of pJL003	ATAATTTTGTTTAACTTTAAGAAGGAGATATACCAATGGGCAGCAGCTGGTCT
oJL010	Reverse primer for generation of pJL003	TCCCATCTGCTAGGACATCATCAATGTCATAGTAACCCATGGATTGGAAGTACAGG
oJL020	Forward primer for generation of nucleosome flanked ARS1	CGATAGAACTCGGGCCGCCCTGGAGAATCGCGGTGCCG
DNACSR1	Reverse primer for generation of nucleosome flanked ARS1	CCTGCACCCCAGGGACTTGAAGTAATAAGGAC
oJL019	Forward primer for generation of 168bp M.HpaII ARS1	ATATATCC*GGCCTGTATTTTACAGATTTTATGTTTAGATCTTTTATGC
oJL040	Reverse primer for generation of 168bp M.HpaII ARS1	AGGGCGCC*GGAACTGGGAAAATAGCAAATTTCGTCAAAAAATGC
oSSH047	Forward primer for generation of ARS1 copy 1	GAATTGACGCGTATTGGGATTCAGCTCCGGATGGTCCATTTTACAGATTTTATGTTT
oSSH048	Reverse primer for generation of ARS1 copy 1	ACCGCTCGTCACAGTTGAACGTTATTACTGAGTAGTATTTATTTAAGTAT
oSSH049	Forward primer for generation of ARS1 copy 2	GTTCAACTGTGACGAGCGGTAGCAAAAGGCCAGGAACCGT
oSSH050	Reverse primer for generation of ARS1 copy 2	CCGCCTGTTGGATATCTGATGTTATTACTGAGTAGTATTT
oSSH051	Forward primer for generation of ARS1 copy 3	ATCAGATATCCAACAGGCGGAGCAAAAGGCCAGGAACCGT
oSSH052	Reverse primer for generation of ARS1 copy 3	TTCTGTAAGGATCCGTATTAGTTATTACTGAGTAGTATTT
oSSH053	Forward primer for generation of ARS1 copy 4	TAATACGGATCCTTACAGAAAGCAAAAGGCCAGGAACCGT
oSSH054	Reverse primer for generation of ARS1 copy 4	TTTCCAAGCTTATCCCCTGAGTTATTACTGAGTAGTATTT
oSSH055	Forward primer for generation of ARS1 copy 5	TCAGGGGATAAGCTTGGAAAAGCAAAAGGCCAGGAACCGT
oSSH056	Reverse primer for generation of ARS1 copy 5	GGCCTTGAATTCACATGTTCGTTATTACTGAGTAGTATTT
oSSH057	Forward primer for generation of ARS1 copy 6	GAACATGTGAATTCAAGGCCAGCAAAAGGCCAGGAACCGT

oSSH063	Reverse primer for generation of ARS1 copy 6	GCTCGGCGCGCCATTGGGATTCAGCTCCGGATGGTCCGTTATTACTGAGTAGTATTTATT
oSSH038	Primer for amplification of M.HpaII array	TCAGCTCC*GGATGGTCC
oMG25	Synthetic DNA fragment (geneart) used to generate pMG39 plasmid using MluI /XbaI restriction sites	ACGCGTCACTGGGGTGGTGACAAGAAGAACAGGA<br>GTCTTCCCTCAATTAAAATATGTCAAATTCAATT<br>GTTTGAAATGTGGCTCCATTTTGGGCCCATTTTT<br>TCAAGATTCTAATGAAGAAATTAGAATCTCATTC<br>TGTACAAACTGCAAATCAAAAGGTCCCTTTAGAG<br>TCAATGGAGAAAAAACTGTGTACCGAAATTATCA<br>AAGGGTTACGCTCCAGGAAGCTCCCGGAACCGTT<br>CCTCCAGGCCGTCTACCAAGACATAGAGAAGTCA<br>TTTTGTTGGCGGATTTGGTAGATGTATCCAAGCC<br>AGGTGAAGAGGTAGAAGTTACCGGCATCTACAAA<br>AATAACTACGATGGTAACTTGAATGCAAAGAACG<br>GATTCCCCGTTTTTGCAACAATTATCGAGGCAAA<br>TTCTATAAAAAGAAGAGAGGGCAATACAGCTAAC<br>GAAGGCGAAGAAGGGTTGGATGTTTTCAGTTGGA<br>CTGAAGAAGAAGAACGTGAATTTAGAAAGATTTC<br>TAGGGATCGTGGTATAATTGATAAAATTATATCA<br>TCGATGGCACCGTCTATCTATGGTCATAGAGATA<br>TTAAAACTGCAGTCGCGTGCTCATTATTTGGAGG<br>TGTTCCAAAAAACGTCAATGGAAAACATTCTATT<br>CGTGGTGATATCAATGTGTTATTATTAGGTGATC<br>CAGGTACTGCCAAATCTCAAATCTTAAAATACGT<br>CGAGAAAACAGCGCATAGAGCGGTCTTTGCAACT<br>GGTCAGGGTGCTTCGGCTGTCGGTCTGACAGCAT<br>CCGCTAGAGCAGATGCTATTACTGCAGAAGCGAC<br>CTTAGAAGGGGGGGCGCTAGTATTGGCTGATAAG<br>GGTGTTTGTTTAATTGATGAATTCGATAAGATGA<br>ACGATCAGGATCGTACATCTATTCATGAGGCTAT<br>GGAACAGCAAAGTATTTCCATTTCCGCGGCCGGT<br>ATTGTTACTACATTACAAGCGCGCTGCTCAATTA<br>TTGCTGCGGCAAATCCTAATGGTGGTAGATATAA<br>TTCAACCTTGCCTTTAGCTCAGAATGTTAGTTTG<br>ACCGAGCCTATTCTGTCTAGA
oMG27	Synthetic DNA fragment (geneart) used to generate	CGTACGCTGGACGTCATTCTTCGAGGTGATAGTG<br>TCGAAAGAGCCAAGCCAGGTGACCGTTGCAAATT<br>TACGGGTGTGGAAATTGTAGTACCTGATGTTACA

	pMG43 using BsiWI/ SphI restriction sites	CAATTGGGGCTACCAGGTGTGAAGCCAAGTTCAACATTAGATGAGGAGGGTATTTCGAAGACTACTGAAGGTTTGAATAGTGGTGTTACTGGTTTACGCTCTCTTGGTGTTCGTGATTTGACATACAAGATTAGCTTTCTGGCATGC
oMG28	Synthetic DNA fragment (geneart) used to generate pMG44 using BsiWI/ SphI restriction sites	CGTACGCTGGACGTCATTCTTCGAGGTGATAGTGTCGAAAGAGCCAAGCCAGGTGACCGTTGCAAATTTACGGGTGTGGAAATTGTAGTACCTGATGTTGAGGAGGAGGAGGAGCCAGGTGTGAAGCCAAGTTCAACATTAGATACTAGAGGTATTTCGAAGACTACTGAAGGTTTGAATAGTGGTGTTACTGGTTTACGCTCTCTTGGTGTTCGTGATTTGACATACAAGATTAGCTTTCTGGCATGC
oMG17	Primer to verify Mcm2 point mutants by sequencing (GeneWiz)	AAGCTCTTCGCCTTCTCG
oMG18	Primer to verify Mcm2 point mutants by sequencing (GeneWiz)	GGTAGATGTATCCAAGCCAG
oMG21	Primer to to verify Mcm5 point mutants by sequencing (GeneWiz)	GCAAACGAGTCGAATATTGGTG
oMG22	Primer to to verify Mcm5 point mutants by sequencing (GeneWiz)	GTGACGAAGTTCGATGACAG
oMG23	Primer to to verify Mcm6 point mutants by sequencing (GeneWiz)	CGAGCATCTGAAGCAAGATAC
oMG24	Primer to to verify Mcm6 point mutants by sequencing (GeneWiz)	GTGACAAGAACATCTGAGGTC

* denotes 5-Fluoro-2’-deoxycytidine

Supplementary Table 2: Recombinant DNA reagents used in this study.

Plasmid	Designation/characteristics	Selection	Reference
pAM3	Cdc6 purification	Amp	Frigola et al., 2013
pJL003	GINS purification (used in EM assays)	Kan	This study
pFJD5	GINS purification (used for unwinding assay and DNA replication assays)	Kan	Gambus et al., 2009
pGC441	Sld2 purification	Amp	Posse et al., 2021
pMD132	Mcm10 purification	Kan	Douglas et al., 2018
pJL004	M.HpaII purification	Amp	This study
pTM011	H2A/H2B	Amp	Saravanan et al., 2012
pTM004	H3/H4	Spec	This study
pTM005	Nucleosome flanked ARS1 origin	Amp	Miller et al., 2019
pSSH005	6x ARS1 array	Amp	This study
pBS/ARS1WTA	3.2-kb plasmid containing ARS1(used for 616 bp DNA preparation for unwinding assay)	Amp	Marahrens and Stillman, 1992
pJY22	10.6 kb plasmid DNA with ARS1 (used for DNA replication assay)	Amp	Yeeles et al., 2017
pJF3	pRS304/Mcm4/Mcm5	Amp Trp1	Frigola et al., 2013
pJF4	pRS305/Mcm6/Mcm7	Amp Leu2	Frigola et al., 2013
pAM38	pRS306/Mcm2_CBP-TEV-Mcm3	Amp Ura3	Coster et al., 2014
pMG39	pMA vector (GeneArt) containing oMG25 between MluI /XbaI restriction sites (used to generate pMG69 plasmid)	Amp	This study/ GeneArt
pMG41	pMA vector (GeneArt) containing oMG26 between BstBI/ NsiI restriction sites	Amp	This study/ GeneArt

	(used to generate pMG52 plasmid)		
pMG43	pMA vector (GeneArt) containing oMG27 between BsiWI/ SphI restriction sites (used to generate pMG53 plasmid)	Amp	This study/ GeneArt
pMG44	pMA vector (GeneArt) containing oMG28 between BsiWI/ SphI restriction sites (used to generate pMG54 plasmid)	Amp	This study/ GeneArt
pMG69	pRS306/Mcm2 6A, CBP-TEV Mcm3 (used to generate yeast strain for Mcm2 6A mutant purification)	Amp Ura3	This study
pMG53	pRS305/Mcm6 2E, Mcm7 (used to generate yeast strain for Mcm6 2E mutant purification)	Amp Leu2	This study
pMG54	pRS305/Mcm6 5E, Mcm7 (used to generate yeast strain for Mcm6 5E mutant purification)	Amp Leu2	This study

Supplementary Table 3: *Saccharomyces cerevisiae* strains used in this study.

Strain	Genotype	Designation	Reference
ySD-ORC	*MAT**a** ade2-1 ura3-1 his3-11,15 trp1-1 leu2-3,112 can1-100*<br>*bar1::Hyg*<br>*pep4::KanMX*<br>*his3::HIS3pRS303/ORC3, ORC4*<br>*trp1::TRP1pRS304/ORC5, ORC6*<br>*ura3::URA3pRS306/CBP-ORC1, ORC2*	ORC purification	Frigola et al., 2013
yAM33	*MAT**a** ade2-1 ura3-1 his3-11,15 trp1-1 leu2-3,112 can1-100*<br>*bar1::Hyg*<br>*pep4::KanMX*<br>*his3::HIS3pRS303/CDT1,GAL4*<br>*trp1::TRP1pRS304/MCM4,MCM5*<br>*leu2::LEU2pRS305/MCM6 MCM7*<br>*ura3::URA3pRS306/MCM2,CBP-MCM3*	Mcm2–7/Cdt1 purification	Coster et al., 2014
ySDK8	*MAT**a** ade2-1 ura3-1 his3-11,15 trp1-1 leu23,112 can1-100,*<br>*bar1::Hyg*<br>*Pep4::KanMX*<br>*trp1::TRP1pRS304/CDC7, CBP-DBF4*	DDK purification	On et al., 2014
yAE88	*MAT**a** ade2-1 ura3-1 his3-11,15 trp1-1 leu2-3,112 can1-100*<br>*bar1::Hyg*<br>*pep4::KanMX*<br>*his3::HIS3pRS303/CBP-TEV-CLB5(D1-100), GAL4*<br>*ura3::URA3pRS306/CKS1, CDC28*	CDK purification	Hill et al., 2020
yJY13	*MAT**a** ade2-1 ura3-1 his3-11,15 trp1-1 leu2-3,112 can1-100*<br>*bar1::Hyg*<br>*pep4::KanMX*<br>*his3::HIS3pRS303/Cdc45iflag2, GAL4*	Cdc45 purification	Yeeles et al., 2015
yJY26	*MAT**a** ade2-1 ura3-1 his3-11,15 trp1-1 leu2-3,112 can1-100*<br>*bar1::Hyg*<br>*pep4::KanMX*	Dpb11 purification	Yeeles et al., 2015

	his3::HIS3pRS303/Dpb11-3xflag (Nat-NT2), GAL4		
yAJ2	*MAT**a** ade2-1 ura3-1 his3-11,15 trp1-1 leu2-3,112 can1-100*<br>*bar1::Hyg*<br>*pep4::KanMX*<br>*ura3::URA3pRS306/Dpb2, Dpb3*<br>*trp1::TRP1pRS304/Pol2, Dpb4-Tev-CBP*	Pol epsilon purification	Yeeles et al., 2015
yAE99	*MAT**a** ade2-1 ura3-1 his3-11,15 trp1-1 leu2-3,112 can1-100*<br>*bar1::Hyg*<br>*pep4::KanMX*<br>*ura3::URA3-Gal1-10 Dpb2 + Dpb3*<br>*Pol2::Pol2-FLAG-NAT*<br>*trp1::TRP1-Gal1-10 Pol2 exo- + Dpb4-CBP*	Pol epsilon exo- purification	Goswami et al., 2018
yTD6	*MAT**a** ade2-1 ura3-1 his3-11,15 trp1-1 leu2-3,112 can1-100,*<br>*bar1::Hyg*<br>*pep4::KanMX,*<br>*his3::HIS3pRS303/SLD3-TCP, GAL4,*<br>*leu2::LEU2pRS305/SLD7*	Sld3–7 purification	Yeeles et al., 2015
yAE95	*MATa ade2-1 ura3-1 his3-11,15 trp1-1 leu2-3,112 can1-100,*<br>*bar1::Hyg*<br>*pep4::KanMX,*<br>*trp1::TRP1pRS304/POL1, POL12,*<br>*ura3::URA3pRS306/CBP-TEV-PRI1, PRI2*	Pol alpha purification	Hill et al., 2020
yAE42	*MATa ade2-1 ura3-1 his3-11,15 trp1-1 leu2-3,112 can1-100*<br>*bar1::Hyg*<br>*pep4::KanMX*<br>*his3::HIS3pRS303/CBP-TOP1+Gal4*	TopoI purification	Yeeles et al., 2017
yAE31	*MATa ade2-1 ura3-1 his3-11,15 trp1-1 leu2-3,112 can1-100*<br>*bar1::Hyg*<br>*pep4::KanMX*<br>*his3::HIS3pRS303/CBP-TEV-RFA1, GAL4,*<br>*ura3::URA3pRS306/RFA2, RFA3*	RPA purification	Yeeles et al., 2015

yJF21	*MATa ade2-1 ura3-1 his3-11,15 trp1-1 leu2-3,112 can1-100*<br>*bar1::Hyg*<br>*pep4::KanMX*<br>*his3::HIS3pRS303/Cdt1,Gal4*<br>*trp1::TRP1pRS304Mcm4, Mcm5*<br>*leu2::LEU2pRS305/Mcm6, Mcm7*	Background strain	Frigola et al., 2013
yAM21	*MATa ade2-1 ura3-1 his3-11,15 trp1-1 leu2-3,112 can1-100*<br>*bar1::Hyg*<br>*pep4::KanMX*<br>*his3::HIS3pRS303/Cdt1,Gal4*<br>*ura3::URA3pRS306/Mcm2, CBP-TEV Mcm3*<br>*leu2::LEU2pRS305/Mcm6, Mcm7*	Background strain	Coster et al., 2014
yAM20	*MATa ade2-1 ura3-1 his3-11,15 trp1-1 leu2-3,112 can1-100*<br>*bar1::Hyg*<br>*pep4::KanMX*<br>*trp1::TRP1pRS304Mcm4, Mcm5*<br>*ura3::URA3pRS306/Mcm2, CBP-TEV Mcm3*<br>*his3::HIS3pRS303/Cdt1,Gal4*	Background strain	Coster et al., 2014
yAE160	*MATa ade2-1 ura3-1 his3-11,15 trp1-1 leu2-3,112 can1-100*<br>*bar1::Hyg*<br>*pep4::KanMX*<br>*trp1::TRP1pRS304Mcm4, Mcm5*<br>*ura3::URA3pRS306/Mcm2, CBP-TEV Mcm3*<br>*his3::HIS3pRS303/Cdt1,Gal4*<br>*leu2::LEU2pRS305/Mcm6 2E, Mcm7*	Mcm6 2E mutant purification	This study
yAE161	*MATa ade2-1 ura3-1 his3-11,15 trp1-1 leu2-3,112 can1-100*<br>*bar1::Hyg*<br>*pep4::KanMX*<br>*trp1::TRP1pRS304Mcm4, Mcm5*<br>*ura3::URA3pRS306/Mcm2, CBP-TEV Mcm3*<br>*his3::HIS3pRS303/Cdt1,Gal4*<br>*leu2::LEU2pRS305/Mcm6 5E, Mcm7*	Mcm6 5E mutant purification	This study

yAE164	*MATa ade2-1 ura3-1 his3-11,15 trp1-1 leu2-3,112 can1-100*<br>*bar1::Hyg*<br>*pep4::KanMX*<br>*his3::HIS3pRS303/Cdt1,Gal4*<br>*trp1::TRP1pRS304/Mcm4, Mcm5*<br>*leu2::LEU2pRS305/Mcm6, Mcm7*<br>*ura3::URA3pRS306/Mcm2 6A, CBP-TEV Mcm3*	Mcm2 6A mutant (Mcm2 V580A/ K582A/ P584A/ K587A/ W589A/ K633A) purification	This study

Supplementary Video 1: Cryo-EM density of the yeast CMGE dimer caught in the act of nucleating origin DNA melting.

Supplementary Video 2: Animation of DH to dCMGE conversion. The molecular morph shows a register shift in the subunit contacts at the MCM dimerization interface. dCMGE formation involves the disruption of an inter-ring Mcm7–Mcm5 contact that reveals a GINS-engagement site on Mcm5. dCMGE formation also involves the formation of a splayed MCM dimer that is kept together by a new Mcm6 dimer interface and by duplex DNA (shown in green). This DNA becomes exposed in between the two helicase rings when dCMGE is formed. Whether the DH to dCMGE transition is symmetric or conformational changes occur sequentially within the two rings remains an open question.

Supplementary Video 3: DH to dCMGE transition involves a change in nucleotide occupancy, which in turn promotes a change in DNA engagement.

Supplementary Video 4: DH to dCMGE transition involves a change in Mcm2 ATPase binding to DNA. While in DH only residue K587 of the h2i pore loop contacts DNA, six pore loop residues engage untwisted DNA in the dCMGE, including four new h2i and one new PS1BH pore loop contacts.

Supplementary Video 5: Mechanism coupling DNA melting and DH separation. DH to dCMGE transition involves the reconfiguration of the Mcm6 wedge insertion. This element moves from the MCM dimerization interface in DH to the inner lumen of the MCM ring in the dCMGE. In doing so, the Mcm6 wedge insertion stabilizes three lagging strand orphan bases that become exposed upon DNA untwisting. Mcm6 residues T423 and R424 move from the outer surface of the MCM ring in the DH to the inner lumen of dCMGE and engage in orphan base stabilization. The structural change in the DNA is first shown in the context of a single MCM hexamer and then across the entire double hexamer.
