# Supplementary material for: Mechanism of replication origin melting nucleated by CMG helicase assembly
Source: Nature. 2022 Jun 15;606(7916):1007–14. doi: 10.1038/s41586-022-04829-4 (PMC9242855; doi:10.1038/s41586-022-04829-4)
Supplement: Supplementary file 2 — Reporting Summary [file 41586_2022_4829_MOESM2_ESM.pdf]

nature portfolio

Corresponding author(s): Alessandro Costa

Last updated by author(s): 25/03/22

Reporting Summary

Nature Portfolio wishes to improve the reproducibility of the work that we publish. This form provides structure for consistency and transparency in reporting. For further information on Nature Portfolio policies, see our Editorial Policies and the Editorial Policy Checklist.

Statistics

For all statistical analyses, confirm that the following items are present in the figure legend, table legend, main text, or Methods section.

n/a	Confirmed	
☐	☒	The exact sample size (*n*) for each experimental group/condition, given as a discrete number and unit of measurement
☐	☒	A statement on whether measurements were taken from distinct samples or whether the same sample was measured repeatedly
☐	☒	The statistical test(s) used AND whether they are one- or two-sided<br>*Only common tests should be described solely by name; describe more complex techniques in the Methods section.*
☒	☐	A description of all covariates tested
☒	☐	A description of any assumptions or corrections, such as tests of normality and adjustment for multiple comparisons
☐	☒	A full description of the statistical parameters including central tendency (e.g. means) or other basic estimates (e.g. regression coefficient) AND variation (e.g. standard deviation) or associated estimates of uncertainty (e.g. confidence intervals)
☐	☒	For null hypothesis testing, the test statistic (e.g. *F*, *t*, *r*) with confidence intervals, effect sizes, degrees of freedom and *P* value noted<br>*Give P values as exact values whenever suitable.*
☒	☐	For Bayesian analysis, information on the choice of priors and Markov chain Monte Carlo settings
☒	☐	For hierarchical and complex designs, identification of the appropriate level for tests and full reporting of outcomes
☒	☐	Estimates of effect sizes (e.g. Cohen's *d*, Pearson's *r*), indicating how they were calculated

Our web collection on statistics for biologists contains articles on many of the points above.

Software and code

Policy information about availability of computer code

Data collection	Gatan DigitalMicrograph and ThermoFisher EPU v2.9
Data analysis	crYOLO v1.7.5, Topaz v0.2.5, MotionCor2, Gctf v1.06, RELION v3.1, cryoSPARC 3.2, UCSF Chimera v1.14, ChimeraX-1.3, COOT v0.9-pre, Phenix v1.19.2, MolProbity web sever, ImageJ v2.0.0, GraphPad Prism v9.2.0, Phyre2 web server, pyem v0.5, PyMOL v2.4.1

For manuscripts utilizing custom algorithms or software that are central to the research but not yet described in published literature, software must be made available to editors and reviewers. We strongly encourage code deposition in a community repository (e.g. GitHub). See the Nature Portfolio guidelines for submitting code & software for further information.

Data

Policy information about availability of data

All manuscripts must include a data availability statement. This statement should provide the following information, where applicable:

- Accession codes, unique identifiers, or web links for publicly available datasets
- A description of any restrictions on data availability
- For clinical datasets or third party data, please ensure that the statement adheres to our policy

Cryo-EM density maps of the CMGE dimer complex has been deposited in the Electron Microscopy Data Bank (EMDB) under the accession number EMD-13988. Cryo-EM density map of the symmetry expanded CMGE monomer has been deposited in the EMDB under the accession number EMD-13978. Atomic coordinates have been deposited in the Protein Data Bank (PDB) with the accession number 7QHS (symmetry expanded CMGE monomer) and 7Z13 (monomer docked into the CMGE dimer map).

Field-specific reporting

Please select the one below that is the best fit for your research. If you are not sure, read the appropriate sections before making your selection.

☒ Life sciences ☐ Behavioural & social sciences ☐ Ecological, evolutionary & environmental sciences

For a reference copy of the document with all sections, see nature.com/documents/nr-reporting-summary-flat.pdf

Life sciences study design

All studies must disclose on these points even when the disclosure is negative.

Sample size	In our negative stain EM experiments, we imaged ATP-dependent CMG assembly, yielding different reaction intermediates. To isolate CMG dimers, we usually collected 100-300 micrographs per condition, these numbers of micrographs were sufficient to eiter allow 2D classificaiton or comparative analysis between MCM mutants.<br><br>To obtain high-resolution structure of the CMGE nucleating origin DNA melting from the mixed population of reaction intermediates in the cryo-EM experiment, ~65.3 K micrographs were collected from two independent grids made from the same CMG assembly reaction. This number of micrographs was sufficient to either allow model building or comparative analysis.<br><br>No statistical methods were used to predetermine sample size.
Data exclusions	For our ReconSil experiments, samples were prepared with reduced concentrations to limit particle crowding and allow the clear identification of single origins of replication. Particles were picked and multiple rounds of 2D classification were performed to isolate particles contributing to the distinct molecular species in our samples. Picked particles that could not be aligned and classified were discarded and therefore were not reconstituted in silico. ReconSiled origins were evaluated and rejected if confident assignment of co-localisation to the same origin could not be made because either, i. the origin was in a region of clustered/aggregated particles or ii. If the origin contained additional particles that had not been 2D classified, and were therefore not overlaid with a 2D class average that would permit confident assignment of the molecular species.<br><br>Negative stain and cryo-EM micrographs with poor staining, ice contamination or entirely lacey carbon, respectively, were excluded. Picked particles that did not align to a distinct class in 2D and 3D (cryo-EM only) were excluded from further analysis. CMGs that were not engaged with pol epsilon were removed from the cryo-EM dataset to yield the best reconstruction of the CMGE dimer complex.
Replication	The cryo-EM dataset of ATP-dependent CMG assembly reaction comprised of a single reaction and two datasets, collected on two independent grids. CMG dimer complex formation in negative stain EM experiments was found to be reproducible across multiple independent sample preparations using different DNA substrates. Details of the number of experimental repeats have been acknowledged in the relevant figure legends. All attempts at data replication were successful. Details of the number of experimental repeats have been acknowledged in the relevant figure legends. All attempts at data replication were successful.
Randomization	For calculation of the resolution of the cryo-EM reconstructions, Fourier shell correlations were calculated using independent halves of the complete datasets, into which the component particles were segregated randomly.
Blinding	Blinding is not relevant for a single particle electron microscopy study such as this.

Reporting for specific materials, systems and methods

We require information from authors about some types of materials, experimental systems and methods used in many studies. Here, indicate whether each material, system or method listed is relevant to your study. If you are not sure if a list item applies to your research, read the appropriate section before selecting a response.

Materials & experimental systems

n/a	Involved in the study
☒	☐ Antibodies
☐	☒ Eukaryotic cell lines
☒	☐ Palaeontology and archaeology
☒	☐ Animals and other organisms
☒	☐ Human research participants
☒	☐ Clinical data
☒	☐ Dual use research of concern

Methods

n/a	Involved in the study
☒	☐ ChIP-seq
☒	☐ Flow cytometry
☒	☐ MRI-based neuroimaging

Eukaryotic cell lines

Policy information about cell lines

Cell line source(s)	S. cerevisiae overexpression strains for CMG assembly and DNA replication proteins have previously described in in

Cell line source(s)	multiple studies across several publications. For clarity to the potential readers and reviewers we have included extensive details in extended data table 4.
Authentication	S. cerevisiae overexpression strains were checked for correct plasmid integration by PCR amplification from extracted genomic DNA.
Mycoplasma contamination	S. cerevisiae overexpression strains were not tested for mycoplasma contamination.
Commonly misidentified lines (See ICLAC register)	No commonly misidentified cell lines were used in this study.
